# The hierarchical levels of information activities in the subject’s psychology and cognition

**DOI:** 10.3389/fpsyg.2026.1884252

**Published:** 2026-09-16

**Authors:** Tianqi Wu, Xiaolong Yang, Kun Wu, Tianyi Ma, Haisha Zhang, Jing Cui

**Affiliations:** 1 School of Humanities and Social Science, Xi’an Jiaotong University, Xi’an, Shaanxi, China; 2 International Center of Philosophy of Information of Xi’an Jiaotong University, Xi’an, Shaanxi, China; 3 Xi’an Conservatory of Music, Xi’an, Shaanxi, China; 4 School of Marxism, Shaanxi University of Technology, Hanzhong, Shaanxi, China

**Keywords:** information science, philosophy of information, subject’s cognition, subject’s information activity hierarchy, subject’s psychology

## Abstract

The subject’s psychological and cognitive activities are important interdisciplinary issues in psychology, cognitive science, and philosophy. The Chinese philosophy of information innovatively provides a philosophical definition of the concepts of subject and object from the dimension of information activity, while also making specific provisions for the cognitive activities of the subject. The subject’s psychology and cognition are an activity of advanced grasping and creation of information. The subject’s information activities have five basic levels. Arranged from lower to higher, they are: the subject’s in-itself information activities, the subject’s intuitive discernment of information, the subject’s memory storage of information, the subject’s subjective creation of information, and the subject’s practice and social realization of information. Higher-level information activities must arise on the basis of lower-level ones. Therefore, the emergence and development of the subject’s psychology and cognition necessarily constitute a highly integrated and complex process of information activities.

## 1 Introduction

In recent years, research on cognition and psychological issues has increasingly tended toward integrative approaches. Frontier explorations across multiple disciplines—including cognitive science, neurophilosophy, psychology, information science, and the Chinese philosophy of information—converge on a core issue: the subject’s interpretation of information and psychological construction is not a single linear process, but rather a complex system characterized by distinct multi-level and highly integrative features. In the area of integrated information research spanning cognition, neural activity, and psychology, Nartallo-Kaluarachchi and colleagues through multi-level irreversibility analysis of brain dynamics, confirmed the existence of nonlinear hierarchical interactions between sensory and cognitive brain regions in information processing (Nartallo-Kaluarachchi et al., 2025). Kagan, starting from information-processing capacity, constructed a quantifiable hierarchical framework ranging from reactive memoryless systems to adaptive, self-regulating agents, thereby providing necessary information-based conditions for identifying “agency” (Kagan et al., 2025). Meanwhile, digital technologies are reshaping the subject’s identity structure, emphasizing a mode of existence based on multi-level information integration. Kovalenko points out that the contemporary subject’s information interpretation has evolved into a distributed, hypertextual form of self-construction (Kovalenko, 2025). Wu, from the perspective of information ontology, summarizes the multiple identities of the subject’s existence, transcendence, and evolution (Wu, 2023). Thus, whether in everyday cognition, technology-assisted activities, or neural activity, the subject continuously interacts with the information environment at different levels and hierarchies. It is precisely this ubiquitous multi-level complex structure that leads us back to the classical division of information forms in the Chinese philosophy of information—in-itself information, for-itself information, and regenerated information. This division reveals a complete evolutionary path of information from objective indirect existence, through the subject’s intuitive grasping, to symbolic creation and re-creation, providing a solid epistemological foundation for understanding the multi-level structure of the subject’s psychology and cognition.

## 2 The informational essence of cognition and psychology

Since Descartes, modern philosophy has closely tied the concept of the “subject” to “consciousness.” Descartes, through his cogito, ergo sum, defined the subject as a “thinking substance” capable of thinking, doubting, understanding, affirming, denying, willing, imagining, and sensing. Kant elevated the subject to a transcendental level, arguing that the subject actively synthesizes sensuous intuitions *a priori* through the categories of understanding, thereby constituting the objects of knowledge. Husserl’s phenomenology, centered on “intentionality,” posits that consciousness is always “consciousness of something.” Sartre further distinguished “being-in-itself” from “being-for-itself,” pointing out that “for-itself” is precisely the essential determination of the subject—it is never what it is, but always transcends itself, negates the given, and creates the future. In this traditional sense, the “subject” is an entity whose essence is consciousness and freedom. Chinese information philosophy inherits and develops this tradition, while also extending it biologically, maintaining that the “subject” specifically refers to beings endowed with consciousness—not limited to human beings. In the 1980s, Chinese information philosophy further specified the subject’s cognitive activities from the dimension of information activities, proposing that “human physiology, psychology, and behavior possess, in essence, a holographic identity” (Wu, 1989a, 1989b, 1989c). It holds that “the subject’s psychology and cognition are an information construction activity that moves relatively within multi-level mediation” and simultaneously an activity of advanced grasping and creation of information (Wu, 1989a, 1989b, 1989c). As this paper is confined to the perspective of Chinese information philosophy and focuses on the informational activity levels of subjective psyche and cognition, it does not attempt to provide an exhaustive review of Western literature.

First, the philosophy of information classifies the forms of information into in-itself information, for-itself information, and regenerated information. It states: “In-itself information is the sign of objective indirect existence, the primitive form of information that has not yet been cognized by the subject. The information field, and the assimilation and dissimilation of information, are two basic forms of in-itself information. For-itself information is the primary stage of subjective indirect existence, the form of intuitive grasping of in-itself information by the subject. It includes two basic forms: the discernment and perception of information, and the recollectable storage of information (sensory memory). Regenerated information is the advanced stage of subjective indirect existence, the form created by the subject. Its basic forms are conceptual information and symbolic information. Conceptual information is new imagery information created by imaginative thinking, while symbolic information is a symbolic representation of for-itself information and image information” (Wu, 1985). Among these, in-itself information refers to objective information forms that do not depend on the subject’s consciousness, while for-itself information and regenerated information belong to the information within the subject’s consciousness.

Since Descartes, the concept of the “subject” in modern philosophy has been closely tied to “consciousness.” Through his cogito ergo sum, Descartes defined the subject as a “thinking substance” capable of thinking, doubting, understanding, affirming, denying, willing, imagining, and sensing. The core marker of the subject is self-consciousness—it not only perceives external objects but can also take its own perceptual activities as objects of reflection. Kant elevated the subject to the transcendental level, arguing that the subject actively synthesizes sensory intuition through innate categories of understanding, thereby constructing objects of knowledge. Here, the core function of the subject lies in synthetic unity and self-consciousness (apperception). Kant emphasized that the “I think” must be able to accompany all my representations; otherwise, these representations would not belong to me. Husserl’s phenomenology, centered on “intentionality,” holds that consciousness is always “consciousness of something.” Sartre further distinguished between “being-in-itself” and “being-for-itself,” pointing out that the “for-itself” is precisely the essential determination of the subject: it is never what it is, always transcending itself, negating the given, and creating the future; its core characteristics are freedom and the capacity for nihilation.

Summarizing the above classical expositions, the core determinations of the concept of the “subject” in the philosophical tradition can be outlined as: self-awareness (the ability to be conscious of one’s own cognitive activities), intentionality (conscious activities are directed toward objects and endow them with meaning), freedom and negativity (the ability to transcend given situations), and symbolic capacity (the ability to use language and symbols for abstract thinking). The “subject” in this tradition is an entity whose essence is consciousness and freedom.

The Chinese philosophy of information both inherits and develops this tradition. In Wu Kun’s system, “for-itself information” is defined as “the form of information constructed by the subject through perception, identification, and processing,” where “subject” specifically refers to beings possessing the capacity for consciousness. This definition avoids falling into humanism because not only humans but also other living beings, and even artificial intelligence, may become subjects. The object, in turn, is specifically the object of the subject’s consciousness. Subject and object are not completely separate; the subject can also take itself as an object in its corresponding cognitive activities. The Chinese philosophy of information unifies cognitive activities and psychological activities within the informational activities of the subject, achieving an integration and unification of cognition and psychology within the subject.

From this classification of information forms, it can be seen that the subject’s cognitive and psychological activities are forms of for-itself information and regenerated information, representing a high-level process of information activity. This advanced form of information emerges and develops from the self-movement and evolution of in-itself information, which universally exists in nature. Since higher-level information activities arise on the basis of lower-level information activities and, moreover, take lower-level information activities as their foundation for unfolding, the emergence and development of the subject’s psychology and cognition necessarily constitute a highly integrated and complex process of information activities. This highly integrated complexity of cognitive activities simultaneously endows the subject’s own information activities with multi-level, highly complex, and integrated characteristics.

The subject interacts with the environment at different levels and hierarchies. Typically, the subject engages with the environment in threefold identity. The subject includes all agents that receive, process, and act upon information, and the human being is the most common and most complex form of the subject. Taking a human being as an example: first, as a general entity, the human being continuously exchanges information with the external world at the level of in-itself information; second, as a cognitive subject, the human being grasps, processes, and creates information at the level of for-itself information and regenerated information; third, through social practice, the human being transforms the environment and, in the process of transformation, realizes his or her purposive information. In the sense of these three identities, the multi-level, highly complex and integrated characteristics of the human subject’s information activities become fully evident. Moreover, “the subject grasps the object with the subject’s own reference frame as a standard.” Therefore, each subject has its own unique cognitive structure. “The subject, as an internal information constructor, experiences three kinds of information interaction in the process of cognition: first, the information received from the object; second, the information already condensed in the neural structure of the subject; and third, the interference from other information within the subject’s body outside the nervous system” (Wu, 1989a, 1989b, 1989c).

Human information activity constitutes an organically integrated whole, and this whole, at a highly synthetic level, forms the subject’s actual cognitive structure. For a cognitive subject who is concretely engaged in information processing operations, each of their psychological activities is a real, ongoing process of information generation. The possible modality of information in such a generative process depends on the integration of two factors: on the one hand, the stimulation of specific information from outside or inside the body (including within the nervous system), and on the other hand, the background reference of the subject’s internally pre-established cognitive structure of information. Because the different levels of human information activity are universally interconnected and internally unified, when the subject’s cognitive structure of information functions as a background reference, it always operates at a synthetic level of different strata and in some holistic, interrelated manner. The result of this synthetic, holistic, and interrelated mode of operation is that all levels of the subject’s information activity provide a referential background for any single level of information activity; at the same time, this synthetic and holistically interrelated background reference is realized through the universal permeation or interweaving of different levels of information activity into any given level.

From the perspective of the philosophy of information, the subject’s cognitive processes and psychological processes are a series of information activities belonging to one and the same system. The fact that scientific research distinguishes them into different domains—such as philosophical epistemology, psychology, and neuroscience—is due solely to differences in research focus and methodology; the object of study is one and the same complex process of information activity, all part of the subject’s information activity.

In summary, the concrete investigation of the multi-level complex structure of the subject’s information activity thus becomes the most fundamental major issue upon which the system of informational epistemology is constructed. Experimentally, we can design various experimental methods for theoretical verification, including thought experiments and interaction-observation experiments.

## 3 The first level of the subject’s information activities: in-itself information activities

Aksentijevic’s theory of the “human information field” regards subjective consciousness itself as the primary field of information, inquiring into how objective in-itself information enters subjective experience. From Aksentijevic’s standpoint, information is not an objective fact independent of the mind; rather, it is always an internal product of the subjective conscious field—that is, information is essentially subjective. What we usually refer to as “objective information” or the external world is merely content within the subjective conscious field, a derived phenomenon endowed with “objective” status through inter-subjective conventions. The true ontological foundation is the subjective conscious field itself, not some independent objective information (Aksentijevic, 2025). In contrast, the Chinese Philosophy of information endows “objective information” with a fundamental ontological status. Wu Kun fundamentally changes the division of being, arguing that the traditional dichotomy of “matter + spirit” is insufficient to describe the world itself. In the objective world, beyond the material world of “direct existence,” there also exists an “indirect existence” world of in-itself information. This world is constituted by the manifestation of interactive relations among things and possesses the unique property of “objective non-reality.” “Objective information” is precisely the identifier of this world, constituting the true object of the subject’s perception. Wu Kun defines objective information as “the self-manifestation of the mode and state of existence of matter (direct existence).” This indicates that objective information is a manifestation of the material world itself and therefore possesses objectivity. It preserves the structure of the material world by means of “indirect existence.” A classic example is the “moon in water”: the image of the moon as matter (direct existence) appears in water as another moon (indirect existence). This moon in water, as objectively existing information, is both objective and non-real.

The Chinese Philosophy of Information holds that objective information and in-itself information are the same concept, and points out that the in-itself assimilation and alienation of information are the most basic and universal forms of information activity. The ascent of information from in-itself to for-itself presupposes the emergence of an information control and processing system capable of perception. However, any activity of perceptual discernment of for-itself information must first take as its realistic background a corresponding lower-level activity of in-itself information. Every subject, whether a lower animal, a higher animal, or a human being, first interacts with the environment as a general entity. At this level of interaction, both animals and human beings interact with environmental objects through the information field spontaneously generated by themselves, and in the course of this interaction, they continually dissimilate their own information and assimilate environmental information. During this process of in-itself assimilation and dissimilation of information, animals and human beings, like inanimate objects, also engage in certain information-internal regulatory activities that alter their own structure, and such information-internal regulation operates first at the in-itself level.

Taking human beings as an example, the subject’s in-itself information activities exist not only in the interaction between human beings and the environment but also within and between the corresponding components, cells, organs, tissues, and systems inside the human body. Any significant or subtle change in the body’s physiological structure or state is realized through the assimilation and dissimilation of information in interaction with the external environment or within and between different systems, tissues, organs, cells, and components inside the body. Usually, these changes in physiological structure do not necessarily produce a subjective imagery effect of intuitive discernment. After working outdoors for a period, we find that the exposed parts of our skin become darker. The darkening of this part of the skin is an encoding effect of information structure: it condenses, through this change in structure and color, the information of having received relatively strong light exposure. However, the concrete encoding process of this condensed information does not produce a corresponding degree or level of subjective imagery of intuitive discernment; this process proceeds at the purely in-itself level. The circulation of various body fluids and blood, the digestion of food, and the occurrence, development, and disappearance of various pathological changes inside our body usually do not produce a corresponding subjective imagery of intuitive discernment either; otherwise, we would not need to develop the diagnostics of human medicine. In fact, the vast majority of the most basic information activities carried out by human beings in internal and external interactions occur only at the purely in-itself level, and only a very small part of these information processes can be perceived by the human nervous system and thereby ascend to the level of for-itself information activities. Even this small part of information activities that ascend to the for-itself level must first proceed in the mode of in-itself information activities. Generally speaking, the information discernment effect of “internal subjective presentation” occurs only when the information stimulus reaches an individual’s specific perceptual threshold and, moreover, precisely when the corresponding sensory organs, nerves, and brain are in a particular state of alertness.

For example, by integrating experimental data from auditory masking detection and sensorimotor detection, researchers have systematically examined the effect of alertness on near-threshold perceptual decision-making. The experimental results show that as alertness decreases, subjects’ detection sensitivity (d’) and discrimination sensitivity (slope) to near-threshold stimuli significantly decline, the perceptual threshold slightly increases, while the response criterion does not change significantly. Therefore, only when stimulus intensity reaches an individual’s specific perceptual threshold and the nervous system is in a sufficiently high state of alertness can the subjective-conscious information discernment effect be triggered (Xu et al., 2025). This is highly relevant to the Chinese Philosophy of Information’s theories concerning the subject’s perceptual threshold of information, as well as the awakening of higher-level information and the inhibition of lower-level information. At the same time, empirical research on subliminal cognitive mechanisms provides supplementary evidence for the continuity of the transition from “in-itself information” to “for-itself information.” Using a dual-modal experimental paradigm combining vision (presented via virtual reality) and touch (electrical skin stimulation), it was found that although subjects could not explicitly perceive the presence of subliminal stimuli, their accuracy in discriminating stimulus intensity under dual-modal conditions (audiovisual or audiovisual-tactile) was significantly higher than under single-modal conditions, and the dual-modal activity pattern in front oparietal multimodal integration regions was also significantly different from that in single-modal conditions. This demonstrates that even at information levels that are not subjectively felt, the brain can still perform cross-modal “integration” of schemas from multiple sensory modalities (Gemignani et al., 2024). Therefore, it is precisely the vast amount of in-itself information activities below the for-itself threshold that continuously provide the subject with evolutionarily indispensable information preparation and bodily regulation for coping with a complex and changing environment, making it possible for in-itself information activities to serve as the universal informational background for for-itself information activities.

In-itself information activities constitute the most fundamental and basic level of the subject’s information activities. At this level, the two basic forms of in-itself information activity (the information field, and the assimilation and dissimilation of information) are universally present. The subject constantly emits an information field (light, electricity, heat, taste, sound, etc.) that manifests its own existence to the environment; within the subject, different systems, tissues, organs, cells, and even different components within the same cell universally engage in interactions mediated by various forms of information fields (bioelectric fields carrying specific information, chemical transmitter fields formed by the release and diffusion of different kinds of enzymes, fields formed by the release and diffusion of various body fluids and particles, etc.); the subject also constantly receives information from environmental objects in various ways through interaction with the environment. Through interactions mediated by various information fields, the in-itself assimilation and dissimilation of information—that is, the universal construction of various in-itself traces—occurs universally both between the subject and the environment and among different structures and components within the subject.

The activity of the subject’s in-itself information constitutes a higher-level information activity of the subject, establishing the deepest-level, universally informational background, and the foundation of real possibility for the emergence and development of the subject’s cognitive activities. The assimilation and alienation of information can be verified through a sphere collision experiment: a metal ball rolls down a ramp and collides with a plasticine ball. After the two balls collide, a certain indentation appears on the plasticine ball. Without examining the metal ball, we can determine the size and diameter of the metal ball that struck the plasticine ball solely through the indentation. This indicates that an assimilation of information has occurred between the metal ball and the plasticine ball; the plasticine ball carries the structural information of the metal ball. Looking back at the metal ball, the side that contacted the plasticine ball is coated with grease from the plasticine, demonstrating that part of the material structure and the carried information of the plasticine ball have undergone an alienation of information from the plasticine ball itself. That is, the originally unified plasticine ball has now been alienated into two parts, one of which has become assimilated with the structural information of the metal ball. This is a complete manifestation of the assimilation and alienation of information in interaction. Such an information activity process can be extended to all interactive relationships among things.

## 4 The second level of the subject’s information activities: intuitive discernment of information

The subject acquires external information through an information reception system, which in living subjects is generally the nervous system. In the process of assimilating external information (including information from the external environment, various states of bodily activity, and information from the subject’s various behaviors), the human nervous system and brain can transform part of the assimilated information into a form of subjective discernment characterized by “internal subjective presentation”—this is what psychology calls perception. Traditional psychology distinguishes between two forms of information acquisition from the external world: sensation and perception. This distinction originated with structuralist psychology. Edward Bradford Titchener explicitly separated sensation—the direct reflection of the basic attributes of a stimulus—from perception, which is a higher-level process of organizing and interpreting sensory information, arguing that conflating the two is the source of many psychological controversies (Köteles, 2024). Subsequently, Gestalt psychology made important revisions to this distinction, emphasizing the principle that the whole takes precedence over its parts and pointing out that perception is not a simple sum of sensations but an active process by which the brain organizes sensory information holistically. In the contemporary cognitive psychology stage, the sensation-perception distinction has become the basic framework of information-processing theory, and the traditional “sensation-perception” dichotomy is undergoing profound re-examination. On the one hand, predictive processing frameworks represented by predictive coding and active inference have fundamentally reshaped our understanding of perceptual processes: perception is no longer a linear processing of passive sensory input by the brain, but an iterative process in which the brain actively performs probabilistic inference and hypothesis testing on sensory information through internally generated models. Sprevak and Smith point out that the historical trajectory of predictive coding—from an idea about encoding efficiency in the visual system to a general model covering perception, cognition, and motor control—provides a new perspective for understanding the brain’s multi-level information processing mechanisms (Sprevak and Smith, 2023). Heilbron and Chater systematically review the empirical evidence for the two major theories of predictive coding and active inference, concluding that although existing empirical evidence provides only moderate support for predictive coding, it shows significant advantages in explaining behavioral data (Heilbron and Chater, 2023).

On the other hand, the variable cognitive penetrability of perception is challenging the traditionally relatively independent status of perception. Vetter and colleagues have for the first time examined empirical evidence for cognitive penetrability across multiple sensory modalities, including vision, audition, somatosensation, vestibular perception, and chemosensation. Their conclusion shows that most sensory modalities exhibit varying degrees of cognitive penetrability, the extent of which varies depending on the intrinsic multimodal characteristics of the modality, the degree to which it is subject to indirect cognitive influence, and whether hedonic evaluation is an inherent element of perceptual experience. Therefore, discussions of perceptual penetrability require a more modality-specific differentiated perspective (Vetter et al., 2024). Furthermore, using heading perception as a model system, Liu and Zeng reveal the computational principles and neural pathways under the Bayesian optimal integration framework, further highlighting the critical role of dynamic integration and recalibration of multimodal information in the process of perceptual construction (Liu and Zeng, 2025). Consequently, contemporary psychological inquiry into perceptual phenomena has long surpassed the simple dichotomy between sensation and perception. By employing diverse frameworks such as predictive coding, Bayesian inference, and cognitive penetrability, it is now deeply revealing the dynamic, recursive, and multi-level complex relationships between sensory input and psychological construction in the subject’s interpretation of information. This completes the subject’s hierarchical transition from in-itself information to for-itself information. The sensory discernment of information as internal subjective presentation constitutes the second level of the subject’s information activities. At this level, information ascends from in-itself to for-itself. Tracing the evolutionary process of biological subjects, we find that the earliest capacities to develop in information reception were sensation and short-term memory, followed by perception and long-term memory.

The high development and refinement of animal perceptual capacities depend on the evolution of the cerebral cortex itself and also on the emergence and development of higher-level psychological activities. Since the different levels and forms of animal psychological activities form a unified interacting whole, lower-level forms of psychological activity are controlled and regulated by higher-level forms. Thus, the refinement of lower-level psychological activities naturally depends on the development of higher-level psychological activities.

If we examine sensation and perception from the perspective of the philosophy of information, the greatest difference between the two lies in the degree of information discernment. Sensation is merely the discernment of individual, simple information displayed by things; it cannot form a holistic image of a thing. Perception, on the other hand, is the synthesis of the individual, simple information discerned by sensation; through this synthesis, a complete image of the object is presented to the subject. Generally speaking, pure sensation in the strict sense may be directly experienced only in the information discernment processes of lower animals. In higher animals, especially humans, because well-developed perception, memory, and more advanced psychological activities have evolved, pure sensation discernment can no longer be directly experienced. In actual information discernment, it is very difficult for people to separate sensation from perception in their own experience. People often directly grasp objects in the form of perception, with few experiences of pure, isolated sensation. Usually, only when observing perceptual activities with specialized instruments, or only when performing concrete theoretical analyses of psychological levels, can the processes of human sensation and perception be separated and described and studied independently.

Saying that people often grasp objects directly in the form of perception does not mean that human perception can transcend the process of sensation and be achieved directly. In fact, the holistic image of an object formed in the perceptual process is achieved by integrating the individual information obtained through individual sensations. This integrative process depends on two aspects of informational factors: first, the quantity and quality of the individual information obtained through sensation; second, the integration mode and informational reference background that the cognitive subject can provide when integrating sensory information. The integration mode depends on the physiological structural characteristics of the perceptual system and the mode of active activity, while the informational reference background depends on the subject’s internally *a priori* condensed cognitive structure and cognitive pattern. The discernment of information by human perception must never be reduced merely to an additive processing of individual sensory information. Since perception is the integration by the cognitive subject of individual sensory information, this integration necessarily relates both to the information obtained through sensation and to the cognitive subject’s internal cognitive mode and pre-existing information structure. Here, there are two sources of information that contribute to the formation of the perceptual holistic image of the object: one is the external object, and the other is the subject’s internal cognitive mode and information structure. Thus, the information participating in the integration process is multi-faceted and multi-level. From this multi-functional perspective, we agree with and endorse the following propositions: perception is not merely an image formed by direct stimulation from object information; rather, perception is an active, selective, organizational, constructive, and interpretive activity process of the cognitive subject on sensory information. Perception is the process by which the perceiver uses information patterns and frameworks from memory and experience to select, organize, and even reconstruct object information so as to complete the construction of a holistic perceptual image. From this, we can also see the complexity, comprehensiveness, and active nature of the subject’s intuitive discernment of information.

The subject’s intuitive identification of information can be verified through a perceived water cup experiment: Place a water cup on a table. Under sufficient lighting, our naked eyes receive light of different wavelengths and intensities reflected from the cup, and extract the differential information carried by the light, enabling direct observation. This is the simplest sensation. However, to distinguish whether it is a real water cup or a painting of a cup, we need to observe the cup from different angles; we need to approach and touch the cup to perceive tactile information such as different pressures and frictions conveyed by its material structure; we need to construct a further intentional structure regarding the cup to obtain holistic, multi-dimensional perceptual information about the cup; and further, we need to integrate the unique individual information already pre-constructed within the subject—including the understanding and comprehension of the concept of a water cup, as well as distinct memories and emotions associated with the cup—through dynamic integration and recalibration of multimodal information, ultimately constructing a more multi-dimensional abstract information identification process.

## 5 The third level of the subject’s information activities: memory storage of information

The third level of the subject’s information activities consists of memory activities, i.e., the encoding, retention, and reproduction of experiential information to form appearances. The concept of “appearance” traces back to ancient Greece. Plato in *The Republic* touched upon imagery in a metaphorical way, regarding images as “copies” of the real world, while the real world itself was a copy of the “world of Forms”; thus appearances were twice-removed, false copies of truth. Aristotle, however, argued that “thinking is impossible without an image” and defined “phantasia” as the bridge between sensation and thought (Bourlogianni, 2025). In modern times, Hume distinguished between “impressions” and “ideas,” where ideas are essentially appearance, and reduced all complex knowledge ultimately to simple sensory impressions and their images. In the late 19th century, structuralist psychology formally listed appearances as one of the basic elements of consciousness, and Galton initiated empirical research on imagery vividness. At the same time, Brentano systematically articulated the “Vorstellungstheorie,” distinguishing between physical “images” and mental “internal images,” and regarded mental imagery as the foundation of all psychological phenomena (Nanay, 2025). In the first half of the 20th century, behaviorism expelled appearance from psychology in the name of objectivity. It was not until the cognitive revolution of the 1970s that research on appearance experienced a revival. Contemporary neuroscience has further transformed the problem of mental imagery from a descriptive concept into a core scientific question concerning the nature of mental representation and its computational mechanisms.

If considered purely from a genetic perspective, we have good reason to regard human memory activities as being on the same level as human sensory and perceptual activities. We treat memory as the third level of human information activities not from a genetic sense but from the sequential logic of cognitive processes. In human cognition, information is first discerned and then, through further processing, transformed into stored memory content. The sensory discernment of information is the first form of for-itself information, and the sensory storage of information in memory is the second form of for-itself information. It is because of the development of superior memory capacity that higher-level information processes become possible. Consider: if any sensory-discerned information could not be retained in memory for a sufficiently long time, how could we speak of experience for subsequent activities, or prediction of future scenarios, let alone the creative information activity of thinking?

People’s conscious, active, and realistic psychological, cognitive, and behavioral activities have three important characteristics. First, such activities require active monitoring by the subject’s explicit subjective consciousness; therefore, while the cognitive or behavioral activity is unfolding, the subject must maintain the relevant information elements in subjective awareness for a sufficiently long duration. Second, the object information acquired through sensory discernment and the information created internally by the individual’s thinking are continuously reused in subsequent cognitive and behavioral activities. The development of the individual’s cognitive level and the gradual complexification and rationalization of individual behavior are achieved through the continuous accumulation of information at different levels within the individual and the reconstruction of structural patterns. Third, in conscious, active, and realistic cognitive and behavioral activities, people always link the two types of information mentioned above (information from the ongoing activity and previously accumulated information). It is precisely the reference background of previously accumulated information that serves as the evaluation criterion and pattern-matching standard for subsequent cognitive and behavioral activities, while the information from the subsequent activities provides an effective way to test, revise, and develop that previously accumulated informational reference background. Corresponding to these three important characteristics is the human being’s superior capacity for memory storage of information. Without a corresponding level of memory storage capacity, not only would any development of human conscious cognitive and behavioral activities be inconceivable, but even the very existence of human conscious, realistic cognitive and behavioral activities themselves would be inconceivable. Contemporary cognitive neuroscience holds that information at different levels within the individual is gradually accumulated through the memory system, whereby behavioral patterns are reconstructed and rationalized. Working memory, as a bridge linking real-time information processing with accumulated knowledge and experience, continuously provides the necessary reference and evaluation criteria for current conscious activities. Thus, memory is not merely a passive information storehouse but a central mechanism supporting conscious mental activity—it is through the accumulation, reference, and dynamic updating at the memory level that human beings extend the scope of their information activities across time, fundamentally underpinning the evolutionary development and creative transcendence of individual cognition.

Using memory duration and nature as criteria, modern psychology generally classifies memory into three types: sensory memory, short-term memory, and long-term memory. This tripartite model was first proposed by Atkinson and Shiffrin in 1968. Sensory memory generally lasts only a very short time, a fraction of a second; it is one of the most basic and most transient forms of the becoming-for-itself of information, experienced subjectively as the immediate echo of sensorily discerned information. Afterimages are a classic example of sensory memory. Research has shown that, during the pre-stimulus phase, spontaneous arousal fluctuations related to the pupil can increase the initial information availability of sensory memory but at the same time accelerate its decay, indicating that spontaneous arousal states dynamically modulate the temporal characteristics of perceptual persistence (Smith and Busch, 2025).

Short-term memory generally lasts less than 1 minute, or even shorter, and is characterized by the brief retention and reproduction of information following a very short period of perception. Traditionally, short-term memory is considered a limited-capacity window in which conscious information is actively maintained. Some scholars have proposed a highly challenging alternative view: the capacity limitations and duration characteristics exhibited by short-term memory need not be explained by an independent short-term storage system but may instead emerge naturally from the dynamic retrieval processes of long-term memory (Polyn and Woodman, 2025). This implies that the relationship between short-term memory phenomena and the intrinsic mechanisms of the long-term memory system is far more complex and profound than the traditional tripartite model suggests.

Modern memory theory holds that for information to be consolidated in memory, the subject must process it in an appropriate manner. This processing requires a certain amount of time, called the trace consolidation time. Consolidation in this memory process generally depends on repeated repetition and reproduction of the complex encoding of the information to be memorized. The information that is preserved for a long time after such complex encoding, repetition, and reproduction is long-term memory. Information stored in long-term memory is generally retained for more than one minute, up to months, years, or even a lifetime. From the perspective of the philosophy of information, long-term memory is information that, during the becoming-for-itself process of in-itself information, is continuously encoded, eventually selected, and integrated with the subject’s existing knowledge network, thereby forming an inexhaustible invisible background condition for the subject’s subsequent regenerated information activities.

Short-term memory is considered the gateway to long-term memory; bypassing short-term memory to go directly from sensory memory to long-term memory is difficult, perhaps impossible. However, recent research also suggests that the boundaries of the tripartite memory model may be more dynamic and permeable than traditionally believed. Sensory memory and short-term memory are not separated by a sharp break but are driven by a unified dynamic neural resource system, whereby sensory input continues to participate in updating working memory representations in the form of residual information after stimulus presentation ends (Bays and Tomić, 2024). The processing paradigm within short-term memory may itself incorporate the retrieval dynamics of long-term memory; the two seemingly different memory systems may share the same computational principles at the underlying mechanistic level (Polyn and Woodman, 2025).

Whether as a faithful extension of the classical model or as a revolutionary challenge, the in-itself movement of information, the short-term operation of its becoming-for-itself, and its long-term preservation constantly cooperate, interweave, and permeate each other. It is precisely this level-by-level interaction within memory that enables the subject, starting from momentary perceptual experience, through the temporary retention and reproduction in short-term storage, to ultimately achieve abstract, context-sensitive, and transferable storage in long-term memory, thereby continuously unfolding infinite possibilities for the creation of regenerated information and the evolution of thought. The difference between short-term memory and long-term memory is not merely a matter of retention duration. Psychologists sometimes use terms such as working memory or operational memory to refer to short-term memory. In such cases, the information in short-term memory becomes certain activated information elements currently being processed in the subject’s cognitive activities. These information elements can come either from the short-term retention and reproduction of current perceptual information or from the retrieval of certain relevant elements from long-term memory. Thus, short-term memory is sometimes understood as a memory process with a shorter duration than long-term memory, sometimes as a stage leading to long-term memory, and sometimes as certain activated elements of long-term memory that have entered into the current processing operations of realistic cognition.

The subject’s memory storage of information can be verified through a name memory and recall experiment: Write your own name on a piece of paper. Obviously, we can write it because we have remembered the structural information of our name. We can write it with pens of different colors and in different writing styles. No matter what color or font, it is still our name; different information carriers do not affect our memory and recognition of our own name. Each act of writing deepens our memory of our name and reinforces the information trace. At the same time, each act of retrieval is also a process of perception. Therefore, perception and memory are sequential only in a logical sense, not distinguishable as separate processes.

## 6 The fourth level of the subject’s information activities: subjective creation of information

The fourth level of the subject’s information activities is the subjective creation of information, i.e., the activity of regenerated information itself. Conceptual information and symbolic information are the two basic forms of regenerated information. Conceptual information is new image-based information created by the subject through the information processing of thinking. Symbolic information is information created by the subject through the information processing of thinking, whereby subjectively imposed relations on things are used to represent information. The essence of symbols created by the subject’s thinking lies in using one information pattern, originally unrelated, to represent another information pattern through the grafting of human thinking.

If the for-itself information of sensory discernment and memory storage still maintains an intuitive correspondence with the object of cognition, then the regenerated information created by the subject—conceptual information and symbolic information—has already transcended the direct, intuitive correspondence with specific objects. Taking human beings as an example, the regenerated information created by human thinking, in the sense that it is generated through the processing operations of thinking, is first produced in the human brain as a purely indirectly existing form through the design of subjective relations. Unlike for-itself information, which first requires the actual stimulus of corresponding object information and subjectively allows the cognizer to unify the experienced memory image with that corresponding object concretely, regenerated information, as a result of thinking creation, does not have an actual cognitive object directly corresponding to it at the moment of its generation. Of course, the subsequent discovery or creation of a directly corresponding cognitive object through perception and practice is a different process.

In psychology, appearances are often divided into two types: memory representations and creative representation. Memory representations mainly manifest as the reactivation of past experience. “When an individual retrieves the spatial representation of a map from long-term memory, the deployment of spatial attention is qualitatively different from that during visual perception: the former relies on alpha activity modulation in the prefrontal cortex, whereas the latter is dominated by posterior cortical involvement, indicating a qualitative difference in neural processing pathways between images originating from long-term memory and those that are creatively constructed” (Clément and Tallon-Baudry, 2025). Unlike the “echoing” nature of memory representations, creative representation have a unique neural basis, being the product of active construction by the subject in higher-level neural circuits (Li et al., 2023). By recording electroencephalographic activity of healthy participants in real and augmented reality environments, Giannopulu and colleagues revealed the cortico-cortical synchronous neural signature of creative mental imagery, finding that creativity, due to its inherent complexity, cannot be localized to a single brain region but involves a bilateral multi-regional network including the prefrontal cortex and posterior areas (parietal, occipitoparietal, and occipitotemporal regions). This demonstrates at the neural connectivity level that creative imagery involves broader, actively constructed cross-regional information integration (Giannopulu et al., 2022).

Clearly, creative representation and memory representations in psychology are not information activities at the same level. Creative representation are actively constructed by the cognitive subject through the information processing of conscious thinking operations, whereas memory representations are not reconstructed at the level of such conscious thinking operations. A new, more appropriate concept should be used to designate these two types of information activities at different cognitive levels. For this reason, the philosophy of information introduces the concept of “conceptual information” (or “conception”) to refer to what psychology usually calls creative representation, while using the term “conceptions” specifically for memory-based images. At the same time, it regards conceptual information as a form of regenerated information created by imagistic thinking or with the aid of abstract thinking.

Cognitive psychologists regard human beings as “physical symbol systems” capable of information processing. The term “physical” refers to the concrete material system of the human nerves and brain engaged in information processing; the term “symbol” refers to any pattern that this material system can manipulate. In the view of cognitive psychologists, any perceptual impression, memory image, conceptual information (in our sense), as well as language, writing, etc., are all symbols. This theoretical framework, known as the “Physical Symbol System Hypothesis,” was formally proposed in 1976 and long dominated the core fields of cognitive science and artificial intelligence. The hypothesis claims that “a physical symbol system has the necessary and sufficient means for general intelligent action.” Under this theoretical perspective, the essence of an intelligent system is a computational process that operates on symbolic structures (Farkaš et al., 2025). This theory, which broadly generalizes the concept of symbol, is evidently transplanted from the pattern recognition and processing applications of computers. Although such a transplantation is convenient and simple for describing individual psychological activities, it brings about a deficiency that cannot be ignored: the generalization of the concept of symbol does not help in the hierarchical description and study of human information activities, because in this generalized sense the concept of symbol is essentially equivalent to the concept of information. To say “any pattern is a symbol” is equivalent to saying “any pattern is information,” because in the most general understanding—whether in information theory, systems theory, or cybernetics—information is understood as pattern. Thus, saying “any information is a symbol” is also equivalent to the two statements above. In this way, “physical symbol system” becomes “physical information system.” In this generalized sense, the introduction of the concept of symbol loses its own specific qualitative determination and becomes merely an alias for concepts such as pattern or information.

In fact, the definition of the concept of symbol should have a stricter connotation. It should not refer to those information patterns that are directly presented; rather, it should specifically refer to situations in which one information pattern is used to represent another information pattern, and such representation also has a certain subjectively agreed-upon character. Obviously, language, writing, charts, and data are the most developed forms of symbols, but symbols are not limited to these forms. Any specific action or expression of a human being can also be used as a symbol, giving rise to disciplines such as kinesics that specialize in studying such symbols. Under certain specific conditions, the patterns of certain specific objects can also be used to represent events, messages, and intelligence; these object patterns then constitute symbols with corresponding meanings. The symbolic information defined here is used precisely in this sense of artificially imposed pattern representation. Abstract thinking, as the concentrated expression of human beings’ highest cognitive ability, is precisely the process of creating new symbolic information through the logical deduction of symbolic information.

From a genetic perspective, in the animal kingdom, imagistic thinking, which creates conceptual information, occurs earlier than abstract thinking, which creates symbolic information. Therefore, we have reason to say that abstract thinking is a higher form of thinking than imagistic thinking. However, in human beings, especially in the mature human being’s thinking activities, conceptual information and symbolic information, imagistic thinking and abstract thinking, are always interwoven and cannot be completely separated. People can use the manipulation of symbolic information in abstract thinking to create conceptual information, and they can also rely on the imagination of conceptual information created by imagistic thinking to generate and define symbolic information. Here, the transformative and controlling role of higher-level information activities over lower-level information activities is concentrated, as is the relationship of multi-level participation and mutual transformation among the levels of human information activities. Therefore, the concept of symbol essentially transforms and mediates natural information forms, constituting an evolutionary structure from in-itself information, through for-itself information, to regenerated information. A purely symbolic system has no real meaning without the embodied subjectivity of the human being; the information exchange between human beings and the environment ultimately requires the grounding of non-symbolic, physical, multi-sensory experience and interaction with the environment. Only in this grounded way can a truly meaningful, semantically rich conscious life be achieved.

The subject’s creation of information can be verified through an information integration experiment: This is a thought experiment with a high degree of freedom. One can imagine any two images to create a new image—two people’s faces, an apple and a pear, rice and noodles. We possess a powerful information integration ability that allows us to combine these image information elements, which originally have no logical connection, to create new image information. There are many such examples. The unicorn is an image created by combining the body of a horse, the wings of a bird, and the horn of a goat. Moreover, this kind of creation has multiple dimensions, such as different sizes, differences in left–right combinations, or blending the features of the two together. In essence, artificial intelligence’s image generation technology is engaged in information integration and re-creation.

## 7 The fifth level of the subject’s information activities: practice and social realization of the subject’s information

The fifth level of the subject’s information activities is the subject’s practical behavior and the social realization of the regenerated information it creates. The philosophy of information holds that: “Practice is the process in which the purposeful information created by the subject is realized in the object through the implementation of the planning information created by the subject.” “Labor is the purposeful transformation of natural objects by human beings. … It is a process in which a kind of purposeful information created by the subject, through the implementation of another kind of planning information created by the subject as an intermediary, penetrates into the object, transforms into the object’s structure and state, and is realized in the object. In essence, it is a process in which the subject’s purposeful information is transformed into the object’s structural information” (Wu, 1986).

Taking human practical activity as an example, it begins with the subject’s purposefulness and planfulness, which are themselves forms of regenerated information created by human cognition and intelligence. This regenerated information in cognition is transformed into command information that initiates the subject’s behavior; through the nervous system, it activates the human motor organs to act, operate tools, and act upon the object. Cognitive neuroscience provides detailed empirical support for this information transformation framework. Priorelli systematically explored, within the active inference framework, the hierarchical dynamic planning ability of the human brain when modeling complex tasks such as tool use, emphasizing that organisms need to not only understand the functional affordances of object manipulation but also learn the multi-level interactions between self and environment. This is precisely the core computational mechanism by which planning information is hierarchically encoded for implementation before the subject’s behavior begins (Priorelli and Stoianov, 2025). At the level of specific encoding of motor planning, posture-based movement planning theory further points out that the selection of the goal posture precedes trajectory generation; its core idea is to treat the target position as an attractor state rather than a simple movement endpoint. Movement itself is a state-to-state transition from one set of goal states to another. This view precisely characterizes how planning information sets the behavioral process by transforming ultimate goals into a sequence of intermediate sub-goals (Ozana et al., 2025). Therefore, the information transformation in human practical activity is not merely a vague philosophical metaphor but a real process with specific cognitive steps such as neural representation of goal states, uncertainty encoding, prediction error correction, and movement trajectory planning.

Throughout this series of practical processes, the subject’s information consistently plays the role of determining the direction of practice, designing its procedures and methods, and controlling its pace and degree. The information externalized by the subject is the main thread running through the entire process. Through this process, the subject’s purposeful information is finally realized in the object, changing the object’s structure and state and making it a product that conforms to human purposive design. Here, whether at the beginning of practice (purposefulness, planfulness), the process of practice (movement of the subject’s organs, operation of tools, processing and transformation of the object), or the outcome of practice (the transformed object), all bear the significance of the subject’s intelligent information activity. What practical activity itself accomplishes is merely the transformation of the purposeful information in the subject’s cognition into the object’s structural information. The completion of this process directly depends on the implementation of the planning information designed in the subject’s cognition to accomplish this process. Moreover, judgments about how the practical process proceeds, the degree to which the object is transformed, and whether the subject’s purpose is achieved as expected require the subject to grasp the practical process and the object being transformed as objects of its cognition. In this process, the information generated by the practical process and the object itself is the object that the subject captures. By capturing this information, the subject continuously issues new information commands to its motor organs, either allowing the practical activity to continue according to the original plan or making appropriate revisions to the original plan. This continuous information cycle process—subject output information → feedback of information from the object (the practical process, the object being transformed) to the subject → subject output of regulatory information—constitutes the information activity process of practical activity itself.

Although the regenerated information created by the subject does not have a specific, directly existing counterpart at the moment of its creation, as long as this regenerated information conforms to the nature of the objective world and possesses the real possibility of being transformed into a directly existing form, it can be realized through human social practice to create a directly existing object corresponding to it. For example, consider the subjective design of a new building; as long as the design is sound and the construction site, materials, and personnel are available, people can, through the concrete process of construction, build a directly existing building according to the subjective design. Thus, the social realization of the subject’s information is achieved through the mediation of human social practice.

Practice is the social realization of the regenerated information created by the subject, or simply the social realization of the subject’s information. What people achieve through the process of social practice is precisely the transformation of the regenerated information created by the subject (purposeful information) into a directly existing form (the specific structure and state of the object).

The social realization of the regenerated information created by the subject also occurs in another sense, namely, that the regenerated information created by individuals enters into social interpersonal communication. In human society, certain words, concepts, symbols, works of art, myths, scientific fantasies, hypotheses, and theories often originate from the information creation of specific individuals; this information is then expressed and narrated in some way, thereby transforming into information shared by people in society. From the specific perspective that when members of society share information created by others, they inevitably undergo changes in their own physiological, psychological, and behavioral structures, we have reason to provide a unified description of the above two different senses of the social realization of regenerated information created by the subject: both are processes in which the subject’s purposeful information is transformed into the structural information of the object (whether human or thing). Of course, besides human society, there is also extensive information exchange in the societies of other species, and the essence is the same. Animal practical behavior also contains rich information transformation. From precise control of object manipulation to the execution of planned sequences, from attention allocation in social learning to selective preferences in tool use, it is evident that animals, in the process of purposefully transforming the external world, possess information processing and behavior organization abilities that go beyond previous understanding. A study from PNAS examined, from both behavioral and chemical analytical perspectives, the strategy of the assassin *pahabengkakia piliceps* using resin to aid predation. Under resin-free conditions, the *pahabengkakia piliceps*’s success rate in capturing bees was less than 30%; with resin, the success rate approached 90%. Chemical analysis further revealed that the *pahabengkakia piliceps*’s handling of resin enhanced the release of volatile compounds, making it more attractive and stimulating to guarding worker bees. The predator essentially exploited the defensive instinct of the bee colony to “lure” worker bees to the ideal attack position. Similarly, the protective and relocating behavior of the abdopus aculeatus toward coconut shells has long been considered evidence of invertebrate tool use, but recent systematic behavioral tracking has further revealed the highly precise and goal-directed sequential execution of the abdopus aculeatus’s actions (Chen et al., 2025). These studies collectively point to the same conclusion: the basic information structure of practical behavior—namely, the transformation of purposeful information into the object’s structural information via the mediation of planning information—is universal across different levels of life.

Thus, the behavioral structure exhibited by animals in their external practical activities—“goal orientation, strategy formulation, means selection, outcome evaluation, and dynamic adjustment”—is isomorphic with the definition of the essence of practice in the philosophy of information. The fifth level of the subject’s information activities, namely the subject’s practical behavior and the social realization of the regenerated information it creates, is precisely the unfolding and embodiment of this process. Whether it is the complex technical labor of human beings or the various predatory behaviors of animals, in essence they can all be regarded as a universal mode of information activity in which the subject transforms internalized purposeful information through the intermediary encoding and organization of operational planning information, ultimately achieving structural transformation in the object of the external world.

The subject’s practical and social realization of information can be verified through an information realization experiment: The subject’s practical activities involving information cover a very wide range. We can return to the earlier experiment of writing one’s name. Each act of writing one’s name is a practical process. When the subject’s name, as purposive information, is realized on paper through the mediation of the body, this constitutes a practical and social realization. This process can be linked to the entire construction of humanized nature. Whether education, the production of commodities, architecture, or even bombing, all are part of the subject’s practical and social realization of information. This encompasses multiple aspects and dimensions, involving every facet of the subject’s life.

## 8 Dialogue between Chinese philosophy of information theory and frontier thoughts

Since the end of the twentieth century, the field of cognitive science has witnessed a profound theoretical paradigm shift, the core driving force of which is a systematic rebellion against the Cartesian tradition of “intracranial cognition”—that is, the view that confines the mind entirely within the skull and reduces cognition to pure computation as symbolic manipulation inside the brain. One of the most radical beginnings of this shift is the “extended mind thesis” proposed by Clark and Chalmers (1998). In their classic paper “The Extended Mind,” the two authors present their core argument through a thought experiment that has been repeatedly cited to this day—Otto, who relies on a notebook to store information, and Inga, who relies on biological memory. Clark and Chalmers argue that cognitive processes do not occur solely inside the brain; if an external resource is functionally equivalent to an internal cognitive process, then that resource itself should be regarded as a constitutive part of the cognitive process, not merely a causal external stimulus. This argument is summarized as the famous “parity principle”: in the execution of a cognitive task, if an external resource plays a role that is functionally indistinguishable from that of an internal neural process, then we must accord that external resource the same cognitive status as the internal process. In other words, Otto’s notebook is not merely an external aid to his brain; it is a genuine constituent of his cognitive system. Clark further expanded this position in his subsequent monograph *Supersizing the Mind*, explicitly arguing that the active body and its interactions with external artifacts are not merely scaffolding for internal cognition but actually constitute cognition itself (Clark, 2008). However, as the theory developed, researchers gradually identified a shift from “first-wave” to “second-wave” extended cognition: first-wave extended cognition, centered on the functionalist parity principle, was criticized for the “coupling-constitution fallacy”—that is, mistakenly treating the causal coupling relationship between subject and environment as a constitutive relationship. Second-wave extended cognition, promoted by Richard Menary, John Sutton, and others, shifted toward the “complementarity principle,” arguing that external resources are not merely substitutes for internal functions but, through qualitative complementarity with internal functions, enable cognitive operations that the unaided biological brain cannot achieve. For example, long division supported by the use of mathematical notation systems cannot be performed through internal mental arithmetic alone. Menary further proposed the theory of “cognitive practices,” emphasizing that external cultural resources are integrated into the subject’s cognitive system through learned cognitive practices, thereby moving the extended cognition thesis from the functionalist “functional equivalence” argument to the more complex framework of “cognitive integration”.

Beyond the extended cognition thesis, other dimensions of the “4E + S” framework offer their own distinctive theoretical approaches. The embodied cognition dimension, represented by George Lakoff, Mark Johnson, and Shaun Gallagher, holds that cognitive structures are not abstract, amodal symbol systems but are grounded in the body’s sensorimotor systems—the most basic concepts such as causality, time, and number are not purely logical or linguistic constructions but are shaped by bodily experiences of pushing and pulling objects, spatial motion, and the transmission of force. This argument fundamentally challenges the separation assumption in classical cognitive science that “the mind is like computer software and the body is like hardware, proposing instead that “what the legs are for the frog, the brain is for the body”—the mind is not a command center directing the body but a tool serving bodily action (Newen et al., 2018). The embedded cognition dimension emphasizes that cognitive activities are always embedded in specific physical, social, and cultural environments and cannot be understood in isolation from the practical contexts in which they occur. The enactive cognition dimension receives its most systematic exposition in the classic work The Embodied Mind by Varela, Thompson, and Rosch, arguing that cognition is not a passive representation of a pre-given world but an “enaction” or “bringing forth” of the world through sensorimotor coupling (Varela et al., 1991). Enactivism abandons the basic framework of representationalism and instead holds that the cognitive subject and its environment mutually constitute each other through action—cognition is not “knowledge about the world” but “the ability to deal with the world.” The situated cognition dimension places cognitive processes within networks of social, cultural, and historical practices, maintaining that the subject’s cognitive construction is always realized within specific practical fields and interactive activities.

Lambros Malafouris’s Material Engagement Theory (MET) is the most distinctive strand within this lineage. As an important theoretical innovation in the field of cognitive archaeology, MET directly incorporates materiality itself into the cognitive equation. In his 2013 monograph *How Things Shape the Mind: A Theory of Material Engagement*, Malafouris systematically constructs this theoretical framework and proposes two core concepts: “thinging” and “metaplasticity” (Malafouris, 2013). “Thinging” is a carefully crafted neologism that abolishes the subject–object dichotomy, arguing that thinking and making are not two separate sequential processes but are mutually constituted through material engagement—in the process of pottery making, the potter does not first form a complete mental representation and then execute it in the clay; rather, the clay “thinks back” to the potter through resistance, affordances, and unexpected effects, reshaping the potter’s intentions in real time. The cognitive process emerges in the relational space between the hand and the malleable material. “Metaplasticity” describes the intertwining relationship between the brain’s neural plasticity and the plasticity of the material forms we create: unlike simple neural plasticity, which refers merely to the brain’s capacity to reorganize itself through experience, metaplasticity captures the feedback loop in which the creation of material artifacts—tools, symbols, dwellings—alters the cognitive niche, which in turn reshapes neural organization and cognitive capacities across evolutionary and developmental timescales. MET thus rejects the internalist picture presupposed by intracranial cognitivism—that “the brain uses external tools as prostheses”—and insists instead that tools and minds mutually constitute each other. As Malafouris puts it, tools are made and used by the hands, just as hands are made and used by tools, and human intelligence is largely “handmade.” This position has sparked a series of discussions in contemporary cognitive science regarding the internal tensions within 4E cognition. Researchers have pointed out that there is a tension between the body-centered emphasis of embodied cognition and the distributional tendency of extended cognition, and also significant tension between enactivism’s dissolution of the body/environment distinction and extended cognition’s claim of trans-body extension. Some scholars attribute these tensions to a fundamental divergence between “body-centeredness” and “distributionalism”—the former anchoring cognition in the embodied action of living beings, the latter distributing cognition across a broad network that transcends the boundaries of the skin (Malafouris, 2019).

Unlike the above lineage of Western theories, which proceeds from the question of the “boundaries of cognition,” the Chinese Philosophy of Information adopts a fundamentally different philosophical approach: it does not ask “whether cognition can extend beyond the body, but first asks “how being itself should be divided,” and on this basis redefines the original relationship between cognition and information. The fundamental innovation of Chinese philosophy of information lies in its radical transformation of the division of being in philosophical ontology, reshaping the dichotomy of “being = matter + spirit” that had been used by traditional philosophy for thousands of years into a ternary ontological framework of “being = matter + information [in-itself information + mind (for-itself and regenerated information)].” Within this framework, “in-itself information” (i.e., objective information) is defined as “the self-manifestation of the mode and state of existence of matter (direct existence).” In other words, the interactive relations among things constitute, through “self-manifestation,” an information world of “indirect existence.” This world exists alongside the material world but possesses the unique property of being “objective but non-real.” This theoretical construction has profound metaphilosophical significance: it not only carves out an entirely new domain of “objective indirect existence” between the “direct existence” of matter and the “subjective existence” of spirit, but also fundamentally changes the formulation of the “fundamental question of philosophy,” expanding it from the traditional question of “the relation between matter and spirit” to a multidimensional question of “the relations between matter and information, and between information and spirit.” On the basis of this information ontology, Wu Kun further distinguishes three basic forms of information: in-itself information (the information form of objective indirect existence), for-itself information (the subjective information form constructed by the subject through perception, identification, and processing), and regenerated information (the new information form produced by the subject through creative transformation of existing information in thought). There exist universal interrelations, interactions, mutual mappings, and transformations among the three forms of information, and the unity of the three forms is social information. In Wu Kun’s system, “in-itself information” constitutes the deepest-level foundation of the subject’s cognitive activities, providing “the deepest-level, universally informational background, and the foundation of real possibility” for the emergence and development of the subject’s cognitive activities. At the same time, for-itself information and regenerated information constitute the core content of the subject’s subjective cognition and psychological activities. Wu Kun explicitly states that the subject’s cognitive processes and psychological processes are a series of information activities belonging to one and the same system. The fact that scientific research distinguishes them into different domains—such as philosophical epistemology, psychology, and neuroscience—is due solely to differences in research focus and methodology; the object of study is one and the same complex process of information activity (Wu and Brenner, 2014). This position stands in stark contrast to the methodological differentiations among the various theories within the 4E + S framework: although the dimensions of the 4E + S framework converge on the general direction of “transcending intracranial cognition,” they exhibit significant tensions and differences in their specific commitments—from functionalist parity to cognitive integration, from body schema to enactive generation, from situated embedding to the emergence of agency—to the extent that some scholars question whether 4E cognition truly constitutes a unified paradigm. In contrast, Wu Kun’s philosophy of information provides a more thoroughgoing argument for cognitive unity from the ontological level: since cognitive activities and psychological activities are essentially both information activities of the subject, they are already unified ontologically, and any differences exist only at the level of research perspectives, not at the level of the object itself.

When we compare Chinese philosophy of information with the extended cognition thesis, the most significant difference lies in their understanding of the ontological status of “information.” Although Clark and Chalmers’ extended cognition thesis challenges the Cartesian tradition on the question of “whether cognitive processes cross the boundary of the skin,” its theoretical framework remains grounded in functionalism—an external resource can be incorporated into the cognitive system because it is functionally equivalent to internal cognitive processes. This line of argument does not address the more fundamental ontological question of “what information itself is.” In contrast, Malafouris’s Material Engagement Theory, despite sharing a certain family resemblance with Wu Kun’s philosophy in its rejection of internalism and its emphasis on the constitutive role of materiality, focuses on the “causal efficacy” of materiality in the generation of cognition rather than on the ontological status of information itself. Wu Kun’s philosophy of information proceeds from an entirely different level: it first answers the question “what is information,” and then on that basis explains how cognitive and psychological activities unfold as higher-level forms of information activity. Wu Kun’s theory of the assimilation and alienation of information (where assimilation of information refers to the structural transfer and preservation of information between different material carriers, and alienation of information refers to the decomposition and differential development of information) further provides a micro-dynamic explanation for the mechanism of information exchange between subject and object. The third fundamental difference of Wu Kun’s philosophy lies in its specification of the “subject.” Although the Western extended cognition thesis challenges the Cartesian intracranial model of “private mental space,” it does not fundamentally reframe the ontological definition of the concept of the subject. The subject is still understood as an existent capable of cognitive computation, with extended cognition merely expanding the resource boundaries of this existent. Wu Kun’s philosophy of information, by contrast, provides a philosophical redefinition of the subject–object concept from the informational dimension: the subject is defined as an existent possessing the capacity for information processing, and the subject can take itself as an object in cognitive activities; the object is defined as the object of the subject’s consciousness. Subject and object are not two poles that are completely separate, but rather relationally constituted existents that mutually mediate and mutually constitute each other through the for-itself and regenerated activities of information. This redefinition of the subject–object relation not only avoids the subject–object dualistic predicament that persisted in Western philosophy from Descartes to Kant, but also provides an informational solution to the ancient problem of “reflexivity”—how the subject knows itself. From a broader philosophical perspective, the deepest dialogue between Wu Kun’s philosophy of information and the 4E + S cognitive theory lineage may take place at the “metaphilosophical” level. Although the 4E + S framework has successfully directed the philosophy of cognition toward new directions beyond the intracranial model, it does not tell us how being itself should be divided. Wu Kun’s philosophy of information, however, reconstructs the division of being at the metaphilosophical level—it not only explains how cognition is possible, but also explains the ontological foundation that makes cognition possible. This metaphilosophical theoretical innovation makes the Chinese philosophy of information a potential universal philosophical language for the information age, capable not only of engaging in dialogue with other theories within cognitive science, but also of providing systematic solutions across multiple subfields of philosophy, including information ontology, information epistemology, information axiology, and information evolution theory.

Furthermore, the theory of active inference and the theory of predictive processing in psychology are essentially the same core concept unfolded at different levels of abstraction. Both theories originate from the Free Energy Principle first proposed by Karl Friston in 2003. Predictive processing describes “how” the brain operates, while active inference explains “why” it operates in that way. More precisely, active inference is the normative theory of predictive processing, whereas predictive processing is its process theory.

Predictive processing theory holds that we cannot directly perceive the world itself. The brain is always isolated from the external world, receiving only sensory information while continuously making active predictions and inferences. First, the brain possesses an internal “mental model” of how the world works. It constantly predicts incoming sensory signals rather than waiting for those signals to arrive. Second, the perceptual process is essentially a Bayesian inference—combining “prior beliefs (experience)” with “sensory evidence (input)” to infer what is most likely occurring in the external world. Finally, through the process of minimizing free energy, the brain naturally balances “the complexity of beliefs” with “the accuracy of explanations.” The brain tends to find an explanation that accurately accounts for sensory input without being overly complex. When sensory input does not match predictions, the brain updates its internal beliefs to make subsequent predictions more accurate—this is a process of perceptual learning. If updating beliefs cannot eliminate the error, the brain changes the body’s state or the environment through motor commands until sensory input conforms to expectations. Action serves to make the world conform to our predictions. For example, active inference suggests that picking up a water cup does not involve sending complex muscle commands; rather, it sets a predicted state of “the hand being at the location of the cup,” and the motor system automatically executes commands via reflex arcs until the prediction error of “the hand not being at the cup” is eliminated.

Active inference theory also maintains that the cerebral cortex has a hierarchical structure, with higher-level cortices sending “top-down” predictive signals to lower-level cortices. Lower-level cortices compare actual input with predictions, generating “prediction errors,” and transmit these errors “bottom-up” back upward. Neurotransmitters such as dopamine and adrenaline play computational roles in this process.

When comparing this with the theory of information activity levels in the Chinese philosophy of information, the two theoretical frameworks share a rich foundation for dialogue. In terms of commonalities, both theories are grounded in scientific information theory and recognize that the activity of information is fundamental—because the brain is always isolated from the external world, receiving only sensory information. Both theories emphasize that human cognition and psychology possess a hierarchical structure with higher and lower levels, and both attempt to analyze the processes of information interaction between levels, among other shared features.

The differences are also clear. First, the Free Energy Principle, which serves as the foundation for active inference and predictive processing, was formally proposed in 2003, whereas the relevant theories in the Chinese philosophy of information had already formed a coherent system nearly 20 years earlier. Due to a lack of information exchange at the time, they remained unknown to Western academia. Second, the concept of information used in the Chinese philosophy of information is a generalized philosophical concept that can unify the informational concepts across many disciplines in science and philosophy. Information, as indirect existence, possesses an independent nature distinct from direct existence (mass-energy). From the perspective of Chinese information thinking, the brain as a material structure is merely a carrier of information; the human body, organs, neurotransmitters, and so forth are also carriers of information. The Chinese philosophy of information understands material activities and information activities as two sides of the same coin. Therefore, when discussing the theory of levels, one cannot focus solely on the single dimension of the brain or the nervous system. Finally, the Chinese philosophy of information has always possessed an inclusive character of metaphilosophy, capable of accommodating other new scientific and philosophical theories and creating integration and interpenetration among fundamental theoretical ideas. This also demonstrates the uniqueness and originality of the Chinese philosophy of information. It should also be emphasized that the five basic levels of subject information activity proposed by Chinese philosophy of information constitute a theoretical study conducted from a philosophical perspective and currently remain primarily a theoretical framework. In the future, empirical research in fields such as cognitive science and psychology will be needed to further concretize this framework and to compare and validate it against competing models, including the extended mind theory, material engagement theory, embodied cognition theory, predictive processing theory, and active inference theory. This constitutes the next step of the present research.

Regarding experimental design, philosophical research differs slightly from natural science research in its experimental methods. Philosophy primarily employs thought experiments. Thought experiments have both advantages and disadvantages. Their advantage is that they can more easily and quickly integrate abstract and complex information, providing overarching judgments about the possibilities and directions of interdisciplinary research, transcending the limitations of traditional experiments and opening up more possibilities. Their disadvantage is equally clear: thought experiments generally rely on analogical reasoning processes, which carry considerable uncertainty. Further empirical experimental verification is needed to achieve greater scientific rigor and persuasiveness. However, the development of science requires both the certainty of empirical experiments and the exploratory nature of thought experiments. The two complement each other to enable genuine comprehensive scientific development. The Chinese philosophy of information has consistently strived toward the direction of integrated research in philosophy and science.

Finally, as a prospect for this research, with the development of artificial intelligence, the five-level theory of the subject’s information activities can be applied approximately to describe the information processing processes of AI. Although whether AI can be discussed as a subject remains highly controversial in academia, we can still interpret the nature of AI from the perspective of comprehensive and complex information activity processes. This will also be an important research topic for the future of the Chinese philosophy of information. The Chinese philosophy of information is still developing; its relevant theories require further refinement, and integrating new scientific research results will undoubtedly endow it with vigorous academic vitality.

## Data Availability

The original contributions presented in the study are included in the article/supplementary material, further inquiries can be directed to the corresponding author.
